# Admission Shock Index Is an Independent Predictor of In‐Hospital All‐Cause Mortality in Patients With Acute Aortic Dissection and Intramural Hematoma

**DOI:** 10.1002/clc.70333

**Published:** 2026-04-27

**Authors:** Lingbin He, Yequn Chen, Cuihong Tian, Junshuang Tang, Shiwan Wu, Qiongxia Xu, Jiaxuan She, Xuerui Tan, Xin Zhang

**Affiliations:** ^1^ Department of Cardiology First Affiliated Hospital of Shantou University Medical College Shantou Guangdong China; ^2^ Clinical Medical Research Center First Affiliated Hospital of Shantou University Medical College Shantou Guangdong China; ^3^ Laboratory of Molecular Cardiology First Affiliated Hospital of Shantou University Medical College Shantou Guangdong China; ^4^ Human Phenome Institute of Shantou University Medical College, Guangdong Engineering Research Centre of Human Phenome Chemistry and Chemical Engineering Guangdong Laboratory Shantou Guangdong China; ^5^ First Affiliated Hospital of Shantou University Medical College Shantou Guangdong China

**Keywords:** aortic dissection, hospital mortality, intramural hematoma, shock index

## Abstract

**Background:**

Acute aortic dissection (AD) and intramural hematoma (IMH) are associated with high mortality, necessitating reliable early risk prediction. The shock index (SI) is a potential prognostic marker in critical care, but its value in AD/IMH remains unclear. This study evaluated the association between admission SI and in‐hospital all‐cause mortality.

**Methods:**

This single‐center retrospective cohort study included 1250 patients with acute AD/IMH, stratified by an optimal SI cut‐off of 0.6 determined by ROC analysis. Kaplan‐Meier curves and Cox proportional hazards models were used to assess the relationship. Subgroup analyses were also conducted to confirm the consistency of the main findings.

**Results:**

The 30‐day cumulative in‐hospital all‐cause mortality was significantly higher in the SI ≥ 0.6 group than in the SI < 0.6 group (Total: 25.7% vs. 14.4%, *p* < 0.001; Stanford A: 35.5% vs. 25.2%, *p* < 0.001; Stanford B: 13.4% vs. 4.8%, *p* < 0.001). An SI ≥ 0.6 was independently associated with increased in‐hospital mortality (adjusted hazard ratio (aHR) 1.67, *p* = 0.004), consistent across Stanford A (aHR 1.52, *p* = 0.038) and Stanford B (aHR 2.57, *p* = 0.014) subgroups. Furthermore, the association was stronger among patients managed without surgery or thoracic endovascular aortic repair (TEVAR) (Total: aHR 2.02, *p* < 0.001; Stanford A: aHR 1.77, *p* = 0.009; Stanford B: aHR 3.30, *p* = 0.004).

**Conclusion:**

An admission SI ≥ 0.6 is independently associated with increased in‐hospital all‐cause mortality in acute AD/IMH, particularly among those managed without surgery/TEVAR. Admission SI may serve as a simple, rapid, and valuable tool for early clinical risk stratification.

## Graphical abstract

An admission shock index (SI) ≥ 0.6 is a simple, rapid predictor of higher in‐hospital mortality in patients with acute aortic dissection and intramural hematoma. This early risk stratification tool is especially valuable for identifying high‐risk patients managed with conservative treatment.

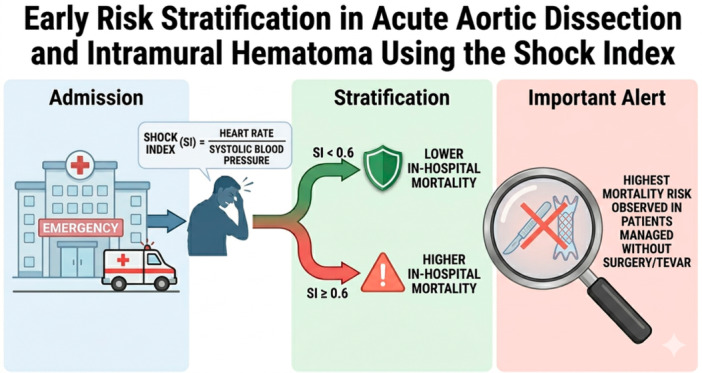

## 1 Introduction

Acute aortic syndrome (AAS) represents a spectrum of life‐threatening vascular emergencies associated with high mortality rates, including aortic dissection (AD), intramural hematoma (IMH), penetrating atherosclerotic ulcers (PAU) and aortic rupture [1, 2]. Among these pathological entities, AD and IMH represent the majority of cases, with AD accounting for approximately 80% and IMH for 15% of AAS presentations [1]. Evidence from Bossone et al. elucidates a J‐curve relationship between systolic blood pressure (SBP) and in‐hospital mortality in acute AD patients [3]. Both extreme hypertension (SBP > 180 mmHg for Stanford A AD and > 200 mmHg for Stanford B AD) and profound hypotension (SBP ≤ 100 mmHg for Stanford A AD and ≤ 80 mmHg for Stanford B AD) are associated with increased mortality risk, while SBP ≤ 80 mmHg emerges as an independent predictor of in‐hospital mortality [3]. Furthermore, heart rate has been identified as an independent prognostic indicator in acute AD by Zhou et al. and Chen et al., demonstrating its prognostic significance in both short‐term and long‐term mortality [4, 5]. Current clinical management guidelines uniformly emphasize stringent hemodynamic control as the cornerstone of initial treatment for acute AD and IMH [2]. Both blood pressure and heart rate are intrinsically linked to AD through their physiological interplay [3, 6]. In hypovolemic states or shock, compensatory tachycardia typically occurs concomitant with hypotension [7]. Therefore, integrated assessment of heart rate and blood pressure may provide enhanced risk stratification capabilities and guide therapeutic strategies for these high‐risk vascular conditions.

The shock index (SI), defined as the ratio of heart rate (beats per minute) to SBP (mmHg), has emerged as a valuable indicator for assessment of severity in hemorrhagic patients. Its proportional increase aligns with progressive reductions in circulating blood volume, making it a critical marker for hemodynamic instability [8, 9]. Recent studies further highlight its utility beyond trauma, demonstrating significant associations between elevated SI and adverse outcomes in conditions such as ruptured abdominal aortic aneurysm [10], stroke [11, 12], acute myocardial infarction [13], pulmonary embolism [14, 15] and severe sepsis [16, 17]. However, the association between SI and clinical prognosis in patients with AAS remains unclear. To this end, this study aimed to evaluate the association between admission SI and in‐hospital all‐cause mortality among patients with acute AD/IMH.

## 2 Materials and Methods

### 2.1 Study Population

**Figure 1:**
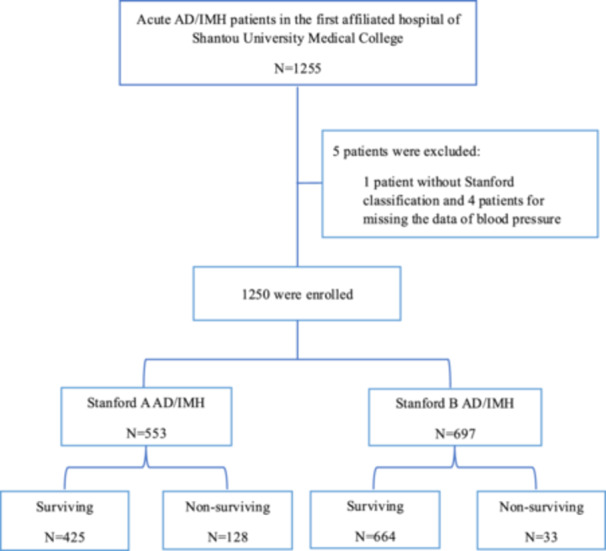
Diagram of screening acute AD/IMH patients. AD, aortic dissection; IMH, intramural hematoma.

This single‐center retrospective cohort study included 1255 consecutively Chinese patients diagnosed with acute AD/IMH [18] admitted between January 2015 and December 2020 Diagnoses were confirmed via computed tomography angiography (CTA) of the aorta. Based on CTA findings, participants were stratified into Stanford A AD/IMH, defined by the presence of an intimal tear or hematoma involving the ascending aorta, and Stanford B AD/IMH, characterized by tears or hematomas localized distal to the origin of the left subclavian artery in the descending thoracic aorta [19]. Demographic and clinical characteristics contained sex, age, SBP, diastolic blood pressure (DBP) and heart rate at admission, history of hypertension and diabetes mellitus, presence of ascending aorta or aortic arch replacement surgery or thoracic endovascular aortic repair (TEVAR), smoking status, serum creatinine (Scr), hemoglobin (HGB), uric acid (UA), total cholesterol (TC), triglycerides (TG), low‐density lipoprotein cholesterol (LDL‐c) and hospitalization time (follow‐up to the 30th day). Renal insufficiency was defined as Scr ≥ 133 μmol/L [20]; hyperlipidemia as TC ≥ 5.2 mmol/L, TG ≥ 1.7 mmol/L or LDL‐c ≥ 3.4 mmol/L [21, 22]; hyperuricemia as UA ≥ 420 μmol/L in males or ≥ 360 μmol/L in females [23, 24]; and moderate anemia as HGB < 90 g/L [20]. Five patients were excluded due to incomplete data: four for lacking blood pressure records, and one for missing the Stanford classification. Consequently, a total of 1250 patients including Stanford A AD/IMH of 553 and Stanford B AD/IMH of 697 were enrolled in the statistical analysis (Figure 1). After admission, all patients received guideline‐directed optimal therapy. This study received ethical approval from the Research Ethics Committee (Approval No. B‐2020‐195). All data collection and analyses were performed anonymously to ensure participants confidentiality. Given the retrospective nature of the study, the informed consent was waived.

### 2.2 Endpoint

The primary endpoint of this study was in‐hospital all‐cause mortality within 30 days. Hospital records were reviewed to identify all‐cause death events among acute AD/IMH patients who died during their hospital stay, with the longest follow‐up period extending up to 30 days.

### 2.3 Statistical Analysis

**Figure 2:**
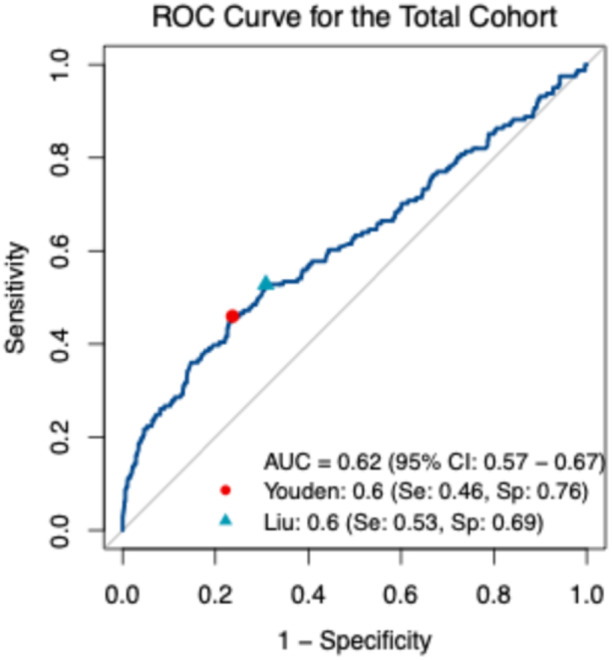
ROC curve for grouping. The AUC was 0.62 (95% CI: 0.57–0.67). The red circle indicates the optimal cut‐off value of admission SI determined by the Youden index (0.6), which yielded a sensitivity of 0.46 and a specificity of 0.76. The blue triangle indicates the cut‐off value of admission SI determined by the Liu index (0.6), with a sensitivity of 0.53 and a specificity of 0.69. AUC, area under the curve; CI, confidence interval, ROC, receiver operating characteristic; Se, sensitivity; SI, shock index; Sp, specificity.

For predicting the in‐hospital all‐cause mortality of acute AD/IMH, participants were stratified into two groups (the SI ≥ 0.6 group and the SI < 0.6 group) based on optimal cut‐off value of admission SI, determined by calculating the maximum Youden index from the Receiver Operating Characteristic (ROC) curve [25]. To confirm the robustness of this threshold, the Liu method was applied as a sensitivity analysis (Figure 2). Continuous variables were assessed for normality using the Shapiro‐Wilk test. Normally distributed continuous variables are presented as mean ± standard deviation (SD) and compared using the Welch Two Sample test. Non‐normally distributed continuous variables are presented as median (interquartile range) and compared using the Wilcoxon rank‐sum test. Categorical variables are expressed as counts (percentages) and compared using the Chi‐square test. For survival analysis, Kaplan‐Meier curves were constructed, and differences were assessed via the log‐rank test. The proportional hazards assumption was verified using Schoenfeld residuals. To identify independent predictors while strictly avoiding overfitting and false positives associated with traditional stepwise selection, we utilized Least Absolute Shrinkage and Selection Operator (LASSO) regularization for automated variable selection [26]. Based on the optimal tuning parameter (λ) associated with the minimum cross‐validation error, 10 clinical variables with non‐zero coefficients were selected, including admission SI, age, sex, diagnosis (AD/IMH), Stanford classification (in the total cohort only), treatment strategy (surgery/TEVAR vs. medical management), hypertension, diabetes mellitus, renal insufficiency, and anemia. These selected baseline variables were subsequently incorporated into a multivariable Cox proportional hazards model to calculate adjusted hazard ratios and 95% confidence intervals. Restricted cubic splines (RCS) with four knots were applied to examine potential nonlinear associations between admission SI and in‐hospital all‐cause mortality. Subgroup analyses were used to evaluate the consistency of SI's prognostic influences across clinically relevant indicators strata.

### 2.4 External Validation Cohort

To validate the prognostic reliability and generalizability of the predefined SI cut‐off of 0.6, an independent external validation cohort of patients with AD was derived from the Electronic Intensive Care Unit (eICU) Collaborative Research Database [27]. One author (Lingbin He, Record ID: 73818326) completed the required training and was granted database access. The validation cohort was established by strictly applying the same inclusion and exclusion criteria, along with the predefined categorical SI threshold (≥ 0.6 vs. < 0.6). Missing baseline data were handled rigorously. Variables with more than 20% missing values were excluded, while those with 20% or less missingness were addressed using multiple imputation via predictive mean matching. Five datasets were generated, and one complete dataset was selected for subsequent analyses to minimize bias. The validation performance was comprehensively evaluated using Kaplan‐Meier survival analysis, a fully adjusted multivariable Cox proportional hazards model, and ROC curve analysis to determine the specific sensitivity, specificity, and area under the curve (AUC) for the 0.6 threshold.

All analyses were two‐tailed, with *p* < 0.05 considered indicative of statistical significance. Data were analyzed by using Stata (Version 19.0 SE, Stata Corp, TX, USA) and R (Version 4.5.2, RStudio 2026.01.0 + 5392).

## 3 Results

### 3.1 Baseline Characteristics

Among the 1250 patients included in the study (918 with SI < 0.6 and 332 with SI ≥ 0.6), baseline demographics including median age (62 years, *p* = 0.991) and male predominance (78.2% vs. 74.1%, *p* = 0.126) were well‐balanced between the two groups. However, patients presenting with an SI ≥ 0.6 exhibited profound hemodynamic instability, characterized by significantly lower median systolic (125 vs. 164 mmHg, *p* < 0.001) and diastolic blood pressures (78 vs. 94 mmHg, *p* < 0.001), alongside a notably elevated median heart rate (91 vs. 74 bpm, *p* < 0.001) compared to the SI < 0.6 cohort. This elevated SI group also harbored a significantly greater proportion of severe aortic pathologies, including higher rates of Stanford Type A classification (57.2% vs. 39.5%, *p* < 0.001) and classic aortic dissection over intramural hematoma (77.4% vs. 65.9%, *p* < 0.001). Regarding comorbidities, while the SI ≥ 0.6 group had a paradoxically lower prevalence of underlying hypertension (71.7% vs. 82.1%, *p* < 0.001) and smoking history (54.5% vs. 65.1%, *p* < 0.001), they suffered from significantly higher rates of renal insufficiency (39.8% vs. 27.5%, *p* < 0.001) and hyperuricemia (49.4% vs. 43.0%, *p* = 0.003). Despite rates of surgical or endovascular interventions remaining statistically similar between the two cohorts (39.2% vs. 40.7%, *p* = 0.614), an SI ≥ 0.6 was strongly associated with poorer clinical outcomes, demonstrating a more than twofold increase in mortality (22.3% vs. 9.5%, *p* < 0.001) and a significantly shorter median follow‐up time (10 vs. 13, *p* < 0.001). These prominent trends of hemodynamic compromise, impaired renal function, and increased mortality associated with a high SI were consistently observed across both the Stanford A and Stanford B subgroups, with the notable addition of anemia reaching statistical significance exclusively within the high‐SI Stanford B cohort (9.9% vs. 5.0%, *p* = 0.040) (Table 1).

**Table 1:** Baseline characteristics of the study participants.

	Total			Stanford A			Stanford B		
	SI < 0.6	SI ≥ 0.6	*p*‐value	SI < 0.6	SI ≥ 0.6	*p*‐value	SI < 0.6	SI ≥ 0.6	*p*‐value
	(*N* = 918)	(*N* = 332)		(*N* = 363)	(*N* = 190)		(*N* = 555)	(*N* = 142)	
Sex			0.126^c^			0.28^c^			0.831^c^
Male	718 (78.2%)	246 (74.1%)		265 (73.0%)	129 (67.9%)		453 (81.6%)	117 (82.4%)	
Female	200 (21.8%)	86 (25.9%)		98 (27.0%)	61 (32.1%)		102 (18.4%)	25 (17.6%)	
Age, years			0.778^b^			0.979^b^			0.728^b^
Median (Q1, Q3)	62 (52, 69)	62 (52, 70)		60 (51, 68)	61 (51, 68)		62 (53, 70)	64 (53, 71)	
AD/IMH			< 0.001^c^			0.082^c^			0.021^c^
AD	605 (65.9%)	257 (77.4%)		281 (77.4%)	159 (83.7%)		324 (58.4%)	98 (69.0%)	
IMH	313 (34.1%)	75 (22.6%)		82 (22.6%)	31 (16.3%)		231 (41.6%)	44 (31.0%)	
Stanford classification			< 0.001^c^						
Stanford A	363 (39.5%)	190 (57.2%)							
Stanford B	555 (60.5%)	142 (42.8%)							
Surgery Intervention			0.614^c^			0.150^c^			0.797^c^
None	544 (59.3%)	202 (60.8%)		183 (50.4%)	108 (56.8%)		361 (65.0%)	94 (66.2%)	
Surgery/TEVAR	374 (40.7%)	130 (39.2%)		180 (49.6%)	82 (43.2%)		194 (35.0%)	48 (33.8%)	
SBP, mmHg			< 0.001^b^			< 0.001^a^			< 0.001^b^
Median (Q1, Q3)	164 (146, 186)	125 (110, 141)		160 (29)	122 (26)		170 (150, 191)	128 (115, 145)	
DBP, mmHg			< 0.001^b^			< 0.001^a^			< 0.001^b^
Median (Q1, Q3)	94 (80, 107)	78 (65, 88)		89 (20)	73 (19)		97 (84, 110)	80 (71, 90)	
Heart rate, bpm			< 0.001^b^			< 0.001^b^			< 0.001^b^
Median (Q1, Q3)	74 (65, 82)	91 (80, 102)		72 (61, 81)	90 (80, 103)		75 (68, 84)	92 (81, 101)	
Hypertension			< 0.001^c^			0.001^c^			0.167^c^
No	164 (17.9%)	94 (28.3%)		74 (20.4%)	64 (33.7%)		90 (16.2%)	30 (21.1%)	
Yes	754 (82.1%)	238 (71.7%)		289 (79.6%)	126 (66.3%)		465 (83.8%)	112 (78.9%)	
Diabetes			0.342^c^			0.274^c^			0.391^c^
No	845 (92.0%)	300 (90.4%)		343 (94.5%)	175 (92.1%)		502 (90.5%)	125 (88.0%)	
Yes	73 (8.0%)	32 (9.6%)		20 (5.5%)	15 (7.9%)		53 (9.5%)	17 (12.0%)	
Smoking			0.001^c^			0.004^c^			0.235^c^
No	311 (33.9%)	146 (44.0%)		133 (36.6%)	93 (48.9%)		178 (32.1%)	53 (37.3%)	
Yes	598 (65.1%)	181 (54.5%)		225 (62.0%)	93 (48.9%)		373 (67.2%)	88 (62.0%)	
Missing	9 (1.0%)	5 (1.5%)		5 (1.4%)	4 (2.1%)		4 (0.7%)	1 (0.7%)	
Renal insufficiency			< 0.001^c^			0.001^c^			0.010^c^
Scr < 133 umol/L	626 (68.2%)	174 (52.4%)		227 (62.5%)	91 (47.9%)		399 (71.9%)	83 (58.5%)	
Scr ≥ 133 umol/L	252 (27.5%)	132 (39.8%)		109 (30.0%)	82 (43.2%)		143 (25.8%)	50 (35.2%)	
Missing	40 (4.4%)	26 (7.8%)		27 (7.4%)	17 (8.9%)		13 (2.3%)	9 (6.3%)	
Hyperlipidemia			0.154^c^			0.196^c^			0.830^c^
No	485 (52.8%)	181 (54.5%)		183 (50.4%)	105 (55.3%)		302 (54.4%)	76 (53.5%)	
Yes	338 (36.8%)	103 (31.0%)		110 (30.3%)	48 (25.3%)		228 (41.1%)	55 (38.7%)	
Missing	95 (10.3%)	48 (14.5%)		70 (19.3%)	3.7 (19.5%)		25 (4.5%)	11 (7.7%)	
Hyperuricemia			0.003^c^			0.195^c^			0.023^c^
No	429 (46.7%)	117 (35.2%)		133 (36.6%)	58 (30.5%)		296 (53.3%)	59 (41.5%)	
Yes	395 (43.0%)	164 (49.4%)		160 (44.1%)	91 (47.9%)		235 (42.3%)	73 (51.4%)	
Missing	94 (10.2%)	51 (15.4%)		70 (19.3%)	41 (21.6%)		24 (4.3%)	10 (7.0%)	
Anemia			0.223^c^			0.938^c^			0.040^c^
HGB ≥ 90 g/L	797 (86.8%)	285 (85.8%)		318 (87.6%)	165 (86.8%)		479 (86.3%)	120 (84.5%)	
HGB < 90 g/L	42 (4.6%)	21 (6.3%)		14 (3.9%)	7 (3.7%)		28 (5.0%)	14 (9.9%)	
Missing	79 (8.6%)	26 (7.8%)		31 (8.5%)	18 (9.5%)		48 (8.6%)	8 (5.6%)	
Survival status			< 0.001^c^			0.001^c^			0.001^c^
Surviving	831 (90.5%)	258 (77.7%)		295 (81.3%)	130 (68.4%)		536 (96.6%)	128 (90.1%)	
Non‐surviving	87 (9.5%)	74 (22.3%)		68 (18.7%)	60 (31.6%)		19 (3.4%)	14 (9.9%)	
Follow‐up time(days)			0.001^b^			0.008^b^			0.031^b^
Median (Q1, Q3)	13.0 (5.0, 18.0)	10.0 (2.0, 17.0)		14.0 (2.0, 21.0)	7.0 (1.0, 19.0)		12.0 (7.0, 17.0)	11.0 (4.0, 15.0)	

Abbreviations: AD, aortic dissection; bpm, beat per minute; DBP, diastolic blood pressure; HGB, hemoglobin; IMH, intramural hematoma; Q1, first quantile; Q3, third quantile; SBP, systolic blood pressure; Scr, serum creatinine; SD, standard deviation; SI, shock index; TEVAR, thoracic endovascular aortic repair.

^a^ Welch Two Sample t‐test.

^b^ Wilcoxon rank sum test.

^c^ Pearson's Chi‐squared test.

### 3.2 Association of Admission SI With the Risk of In‐Hospital All‐Cause Mortality

**Figure 3:**
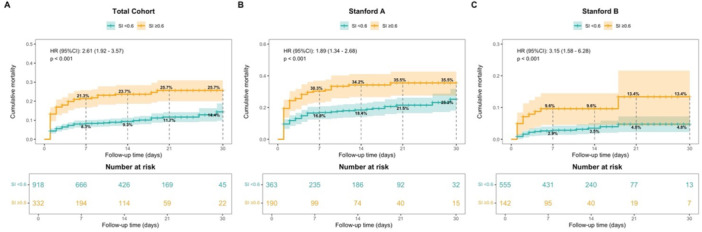
Kaplan–Meier curves of cumulative in‐hospital all‐cause mortality in acute AD/IMH. SI < 0.6 was used as the reference group. A. The total cohort. B. Stanford A AD/IMH. C. Stanford B AD/IMH. AD, aortic dissection; CI, confidence interval; HR, hazard ratio; IMH, intramural hematoma; SI, shock index.

**Figure 4:**
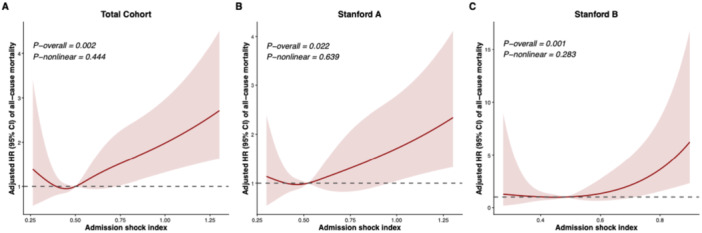
Nonlinear relationship between SI and in‐hospital all‐cause mortality risk of acute AD/IMH evaluated by RCS. All LASSO‐selected covariates including age, sex, diagnosis (AD/IMH), Stanford classification (in the total cohort only), hypertension, diabetes mellitus, renal insufficiency, and anemia, and stratified by surgical intervention, were adjusted in this analysis. The RCS analysis utilized 4 knots placed at the 5th, 35th, 65th, and 95th percentiles of the SI distribution. Solid lines in the figure represent the aHRs, and the shaded regions represent the 95% CIs of aHRs. Dashed lines in the figure represent the reference value of the association, which was set at 1.0. A. The total cohort. B. Stanford A AD/IMH. C. Stanford B AD/IMH. AD, aortic dissection; IMH, intramural hematoma; RCS, restricted cubic splines; SI, shock index.

Kaplan‐Meier curves demonstrated distinct cumulative in‐hospital all‐cause mortalities between SI ≥ 0.6 group and SI < 0.6 group, as illustrated in Figure 3. In the total population, the 7‐day, 14‐day, and 30‐day cumulative mortalities in the SI ≥ 0.6 group were 21.3%, 23.7%, and 25.7%, respectively, whereas these in the SI < 0.6 group were 8.3%, 9.3%, and 14.4%, respectively. In Stanford A AD/IMH, the 7‐day, 14‐day and 30‐day cumulative mortalities in the SI ≥ 0.6 group were 30.3%, 34.2%, and 35.5%, respectively, whereas these in the SI < 0.6 group were 16.9%, 18.4%, and 25.2%, respectively. And in Stanford B AD/IMH, the 7‐day, 14‐day and 30‐day cumulative mortalities in the SI ≥ 0.6 group were 9.6%, 9.6%, and 13.4%, respectively, whereas these in the SI < 0.6 group were 2.9%, 3.5%, and 4.8%, respectively. The log‐rank test revealed significantly higher cumulative mortalities in acute AD/IMH patients with SI ≥ 0.6 than those with SI < 0.6 (*p* < 0.001), no matter what the Stanford classification was. RCS analysis revealed a significant positive linear association between the admission shock index and the adjusted hazard ratio for all‐cause mortality. In the total cohort, the overall association was statistically significant (P‐overall = 0.002), with no evidence of a non‐linear relationship (P‐nonlinear =0.444), indicating that the risk of mortality increases linearly as the shock index rises. This significant linear trend remained consistent across different disease classifications: patients with Stanford A exhibited a significant overall association (P‐overall = 0.022) that was purely linear (P‐nonlinear = 0.639), while patients with Stanford B similarly demonstrated a highly significant overall linear association (P‐overall = 0.001, P‐nonlinear = 0.283) (Figure 4).

Using the variables selected by LASSO, we constructed a multivariable Cox proportional hazards model, which demonstrated that an elevated admission shock index (≥ 0.6) is a significant independent predictor of mortality. In the fully adjusted model (Model 3), which accounted for LASSO‐selected covariates including age, sex, diagnosis (AD/IMH), Stanford classification, hypertension, diabetes mellitus, renal insufficiency, and anemia, while using surgical intervention as a stratification variable, an SI ≥ 0.6 was associated with a 1.67‐fold increased risk of mortality in the total cohort (Hazard Ratio [HR]: 1.67, 95% Confidence Interval [CI]: 1.18–2.37, *p* = 0.004). This independent prognostic value was consistently maintained across clinical subgroups, conferring a significantly increased mortality risk in both the Stanford A cohort (HR: 1.52, 95% CI: 1.02–2.24, *p* = 0.038) and the Stanford B cohort (HR: 2.57, 95% CI: 1.21–5.43, *p* = 0.014) (Table 2).

**Table 2:** Associations of admission shock index with the 30‐day in‐hospital all‐cause mortality in acute AD/IMH patients.

		Model 1		Model 2		Model 3	
Group	Reference	HR (95% CI)	*p*‐value	HR (95% CI)	*p*‐value	HR (95% CI)	*p*‐value
Total Cohort	Admission shock index (< 0.6)	2.61 (1.92–3.57)	< 0.001	2.59 (1.90–3.53)	< 0.001	1.67 (1.18–2.37)	0.004
Stanford A	Admission shock index (< 0.6)	1.89 (1.34–2.68)	< 0.001	1.88 (1.33–2.66)	< 0.001	1.52 (1.02–2.24)	0.038
Stanford B	Admission shock index (< 0.6)	3.15 (1.58–6.28)	0.001	3.11 (1.56–6.22)	0.001	2.57 (1.21–5.43)	0.014

*Note:* Model 1: Unadjusted. Model 2: Adjusted for age and sex. Model 3: Fully adjusted for LASSO‐selected covariates including age, sex, diagnosis (AD/IMH), Stanford classification (in the total cohort only), hypertension, diabetes mellitus, renal insufficiency, and anemia, and stratified by surgical intervention.

Abbreviations: AD, aortic dissection; CI, confidence interval; HR, hazard ratio; IMH, intramural hematoma.

### 3.3 Subgroup Analysis

**Figure 5:**
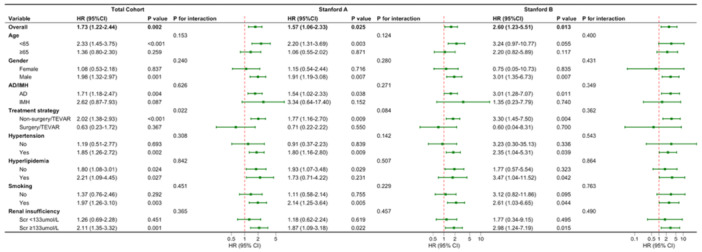
Subgroup analysis of the association between SI and in‐hospital all‐cause mortality in patients with acute AD/IMH. All LASSO‐selected covariates including age, sex, diagnosis (AD/IMH), Stanford classification (in the total cohort only), hypertension, diabetes mellitus, renal insufficiency, and anemia, and stratified by surgical intervention, were enrolled in this analysis. AD, aortic dissection; CI, confidence interval; HR, hazard ratio; IMH, intramural hematom; SI, shock index.

Subgroup analysis demonstrated that the adverse prognostic association between an elevated shock index and increased all‐cause mortality remained highly consistent across most prespecified clinical stratifications (Figure 5). No significant interactions were observed across subgroups including age (< 65 or ≥ 65 years), sex, disease classification (AD or IMH), hypertension, hyperlipidemia, smoking status, and renal function (all *p* for interaction > 0.05). However, a significant interaction was identified between the shock index and treatment strategy in the total cohort (*p* for interaction = 0.022). Specifically, the sharply increased mortality risk associated with a high shock index was predominantly concentrated in patients receiving conservative management (HR: 2.02, 95% CI: 1.38–2.93, *p* < 0.001). Conversely, this risk was significantly mitigated in patients undergoing surgery/TEVAR (HR: 0.63, 95% CI: 0.23–1.72, *p* = 0.367). Notably, while the formal interaction tests for treatment strategy did not reach statistical significance within the individual Stanford A (*p* for interaction = 0.084) and Stanford B (*p* for interaction = 0.362) cohorts, a clear and clinically meaningful divergence persisted. In both anatomical subgroups, an elevated shock index conferred a significantly higher mortality risk strictly among conservatively managed patients (Stanford A: HR 1.77, *p* = 0.009; Stanford B: HR 3.30, *p* = 0.004), whereas this detrimental effect was effectively neutralized in those receiving surgical or TEVAR interventions. This finding highlights the critical clinical significance of an elevated shock index in conservatively managed patients and suggests a potential survival benefit of timely interventional strategies in this high‐risk population.

### 3.4 Validation in the External eICU Cohort

A total of 535 patients with AD from the eICU database were included for external validation, comprising 263 (49.2%) patients with admission SI < 0.6 and 272 (50.8%) with admission SI ≥ 0.6. Baseline characteristics of the external cohort are detailed in Table S1. Consistent with findings from the primary cohort, Kaplan‐Meier analysis demonstrated that patients with an admission SI ≥ 0.6 had a significantly higher 30‐day cumulative in‐hospital all‐cause mortality risk compared to those with an SI < 0.6 (34.1% vs. 20.7%; log‐rank *p* = 0.002) (Figure S1).

Following rigorous variable selection to prevent overfitting, a single comprehensive multivariable Cox model was developed adjusting for age, sex, Stanford classification, hypertension, diabetes mellitus, renal insufficiency, and anemia after stratifying by surgical intervention. In this fully adjusted model, an admission SI ≥ 0.6 remained a strong independent predictor of 30‐day in‐hospital all‐cause mortality (HR 1.91, 95% CI: 1.10–3.33, *p* = 0.022) (Table S2). Furthermore, external validation ROC analysis directly assessing the SI ≥ 0.6 threshold demonstrated a sensitivity of 0.74, a specificity of 0.53, and an AUC of 0.63 (95% CI: 0.58–0.69) (Figure S2).

## 4 Discussion

The present study aimed to assess the predictive utility of SI for in‐hospital all‐cause mortality in patients with acute AD/IMH. The results demonstrated that an SI threshold of ≥ 0.6 was significantly associated with an increased risk of in‐hospital all‐cause mortality, irrespective of the Stanford classification. In addition, compared with patients undergoing surgery or TEVAR, non‐surgical/TEVAR patients with a shock index ≥ 0.6 exhibited a significantly higher risk of in‐hospital mortality in cases of acute AD/IMH, regardless of the Stanford classification. Collectively, these findings suggest that elevated SI may serve as a valuable and independent predictor of in‐hospital all‐cause mortality in patients with acute AD/IMH.

AD and IMH remain life‐threatening emergencies with high early mortality. In our cohort, in‐hospital all‐cause mortality was 23.1% (128/553) for Stanford A and 4.7% (33/697) for Stanford B, slightly lower than historical reports [28, 29, 30, 31, 32], likely reflecting improvements in rapid imaging, multidisciplinary care, and access to both medical and interventional therapies.

Kaplan‐Meier analyses demonstrated that cumulative in‐hospital mortality increased predominantly within the first week of admission, with the rise most pronounced in patients presenting with admission SI ≥ 0.6. This emphasizes the critical need for early risk stratification to identify high‐risk patients who may benefit from closer monitoring and timely intervention. SI is a simple, widely used marker of hemodynamic instability and has proven prognostic value across trauma [33], sepsis [34], hemorrhage [8, 9], and cardiovascular diseases [35, 36]. In acute AD/IMH, instability arises from reduced effective circulating volume, hypotension, tachycardia, and complications such as cardiac tamponade or aortic rupture. Our study confirms that admission SI ≥ 0.6 is an independent predictor of in‐hospital all‐cause mortality, even after adjusting for confounders. Notably, this threshold lies within the conventional normal range (0.5–0.7) [11, 13, 34, 37, 38], highlighting that patients with “borderline” SI may still carry substantial risk. These findings underscore SI's utility for early risk stratification in acute AD/IMH.

Clinically, high‐risk patients who meet surgical or TEVAR indications are closely monitored and receive prompt intervention; however, patients who do not reach surgical criteria often receive less intensive attention, despite still being at considerable risk. Admission SI can serve as a practical marker to identify these conservatively managed patients who warrant closer surveillance and more aggressive medical management.

Focusing on the non‐surgical/TEVAR subgroup, admission SI ≥ 0.6 demonstrated a particularly strong prognostic value. For Stanford A patients managed conservatively, SI ≥ 0.6 was associated with significantly higher in‐hospital mortality (aHR 1.77, 95% CI 1.16–2.70, *p* = 0.009). Although the interaction with surgical status did not reach statistical significance (*p* for interaction = 0.084), the clinical relevance is notable, reflecting the fact that surgical intervention is the most effective strategy for reducing mortality in Stanford A AD/IMH. In this context, SI provides an especially valuable tool for identifying high‐risk patients who may require closer monitoring and intensified medical management. For Stanford B patients, admission SI ≥ 0.6 also predicted increased mortality in non‐surgical/TEVAR subgroup (non‐surgical/TEVAR: aHR 3.30, 95% CI 1.45–7.50, *p* = 0.004; surgical/TEVAR: aHR 0.60, 95% CI 0.04–8.31, *p* = 0.700). Although the *p* for interaction was greater than 0.05 (*p* for interaction = 0.362), the prognostic value of SI appears more pronounced in medically managed patients with Stanford B AD/IMH. These results suggest that surgical or endovascular intervention can partially mitigate the mortality risk associated with elevated SI, whereas in patients treated medically, SI could serve as a strong and independent predictor of early in‐hospital death.

These findings underscore the utility of admission SI as a rapid, cost‐free, and reliable risk stratification tool in patients who are not candidates for surgery or TEVAR. Early recognition of elevated SI in this high‐risk subgroup may inform more intensive monitoring, optimized pharmacologic therapy, and timely consideration of intervention, potentially reducing in‐hospital mortality. Other subgroup analyses confirmed the robustness of SI ≥ 0.6 across sex, age, diagnosis (AD vs. IMH), and common comorbidities such as hypertension, smoking, renal insufficiency, and hyperlipidemia, although interactions with treatment strategy warrant further study.

A major strength of this study is the introduction of an independent external validation cohort from the eICU database. The validation cohort successfully replicated our primary findings, demonstrating that the aHR for mortality (1.91) remains highly significant (*p* = 0.022). This robust external validation crucially addresses concerns regarding the historical nature of the primary dataset, confirming that the prognostic value of an admission SI ≥ 0.6 is not an artifact of older data but a durable, contemporary clinical reality.

While our study benefits from a moderate sample size and has been validated by the external cohort eICU database, several limitations need to be taken into account. The retrospective design may introduce selection bias and unmeasured confounding variables. Furthermore, it is important to note that the predictive accuracy of the SI, as reflected by the AUC (0.62 in the primary cohort and 0.63 in the external validation cohort), is moderate. We emphasize that the SI is not intended to serve as a definitive, standalone diagnostic or prognostic oracle. Instead, its true clinical value lies in its nature as an instantly available, zero‐cost bedside triage parameter. An SI ≥ 0.6 should function as an early warning trigger, prompting clinicians to initiate closer hemodynamic monitoring, expedite advanced imaging, and consider earlier multidisciplinary intervention before overt clinical deterioration occurs.

## 5 Conclusion

Our findings indicate that an elevated admission SI is independently associated with in‐hospital all‐cause mortality in patients with acute AD and IMH. An SI threshold ≥ 0.6 may help identify patients at higher risk, with consistent associations observed across clinical subgroups.

In conclusion, an admission SI ≥ 0.6 is significantly associated with adverse outcomes, particularly among patients managed without surgery or TEVAR. As a simple and readily available clinical marker, admission SI may serve as a useful adjunct to support early risk stratification in acute AD and IMH.

## Author Contributions

All authors have accepted responsibility for the entire content of this manuscript and approved its submission. Xin Zhang and Xuerui Tan designed the study. Lingbin He, Yequn Chen, Cuihong Tian and Junshuang Tang analyzed the data. Lingbin He was major contributor in drafting the manuscript. Xin Zhang and Xuerui Tan revised the article. Shiwan Wu, Qiongxia Xu and Jiaxuan She collected the information of the participants. All authors read the manuscript and approved the final version.

## Ethics Statement

This study received ethical approval from the Research Ethics Committee of the First Affiliated Hospital of Shantou University Medical College (Approval No. B‐2020‐195). The external validation dataset (eICU‐CRD v2.0) is a publicly available, de‐identified database. The establishment and use of the eICU‐CRD were approved by the Institutional Review Boards of the Massachusetts Institute of Technology (MIT) and the eICU Research Institute, and the requirement for individual patient informed consent was waived due to the retrospective and de‐identified nature of the database.

## Conflicts of Interest

The authors declare no conflicts of interest.

## Supporting information

Supporting File 1

Supporting File 2

Supporting File 3

Supporting File 4

## Data Availability

The data underlying this article from our institution are available upon reasonable request due to privacy restrictions. The external validation dataset, the eICU Collaborative Research Database (eICU‐CRD) v2.0, is publicly available. Access to the eICU‐CRD is granted to credentialed researchers who complete the required human subjects training and sign a data use agreement via the PhysioNet repository.
