# Supplementary material for: Admission Shock Index Is an Independent Predictor of In‐Hospital All‐Cause Mortality in Patients With Acute Aortic Dissection and Intramural Hematoma
Source: Clin Cardiol. 2026 Apr 27;49(5):e70333. doi: 10.1002/clc.70333 (PMC13112594; doi:10.1002/clc.70333)
Supplement: Supplementary file 1 — Supporting File 1 [file CLC-49-e70333-s001.docx]

| **Table S1. Baseline characteristics in AD participants from eICU database.** | | | |
| --- | --- | --- | --- |
| **Characteristic** | **Admission Shock Index** | |  |
|  | **SI <0.6** (N = 263) | **SI ≥0.6** (N = 272) | **p-value** |
| Sex, n (%) |  |  | 0.338 *^2^* |
| Male | 172 (65) | 166 (61) |  |
| Female | 91 (35) | 106 (39) |  |
| Age (years), Median (Q1, Q3) | 62 (53, 73) | 64 (54, 72) | 0.154 *^1^* |
| Stanford Classification, n (%) |  |  | <0.001 *^2^* |
| Stanford A | 46 (17) | 87 (32) |  |
| Stanford B | 86 (33) | 64 (24) |  |
| Undefined | 131 (50) | 121 (44) |  |
| Surgical Intervention, n (%) |  |  | 0.008 *^2^* |
| None | 108 (41) | 81 (30) |  |
| Surgery/TEVAR | 155 (59) | 191 (70) |  |
| SBP (mmHg), Median (Q1, Q3) | 146 (130, 164) | 110 (97, 124) | <0.001 *^1^* |
| DBP (mmHg), Median (Q1, Q3) | 74 (62, 87) | 61 (53, 74) | <0.001 *^1^* |
| Heart Rate (bpm), Median (Q1, Q3) | 68 (61, 79) | 84 (74, 97) | <0.001 *^1^* |
| Hypertension, n (%) | 208 (79) | 193 (71) | 0.038 *^2^* |
| Diabetes Mellitus, n (%) | 40 (15) | 36 (13) | 0.596 *^2^* |
| Hyperlipidemia, n (%) | 13 (4.9) | 11 (4.0) | 0.769 *^2^* |
| Renal Insufficiency, n (%) |  |  | 0.129 *^2^* |
| Scr <133 μmol/L | 209 (79) | 200 (74) |  |
| Scr ≥133 μmol/L | 54 (21) | 72 (26) |  |
| Anemia, n (%) |  |  | 0.296 *^2^* |
| HGB ≥90 g/L | 220 (84) | 217 (80) |  |
| HGB <90 g/L | 43 (16) | 55 (20) |  |
| Follow-up Time (days), Median (Q1, Q3) | 5 (3, 9) | 7 (3, 12) | 0.002 *^1^* |
| 30-Day Survival, n (%) |  |  | <0.001 *^2^* |
| Surviving | 245 (93) | 221 (81) |  |
| Non-surviving | 18 (6.8) | 51 (19) |  |
| ^1^ Wilcoxon rank sum test; ^2^ Pearson's Chi-squared test.  ***Abbreviations:*** *AD, aortic dissection; bpm, beat per minute; DBP, diastolic blood pressure; HGB, hemoglobin; Q1, first quantile; Q3, third quantile; SBP, systolic blood pressure; Scr, serum creatinine; SI, shock index; TEVAR, thoracic endovascular aortic repair.* | | | |
