# Supplementary material for: Admission Shock Index Is an Independent Predictor of In‐Hospital All‐Cause Mortality in Patients With Acute Aortic Dissection and Intramural Hematoma
Source: Clin Cardiol. 2026 Apr 27;49(5):e70333. doi: 10.1002/clc.70333 (PMC13112594; doi:10.1002/clc.70333)
Supplement: Supplementary file 2 — Supporting File 2 [file CLC-49-e70333-s002.docx]

| **Table S2. Associations of admission SI with the 30-day in-hospital all-cause mortality in acute AD patients based on eICU database.** | | | | | | | | | |
| --- | --- | --- | --- | --- | --- | --- | --- | --- | --- |
| **Characteristic** | **Model 1** | | |  | **Model 2** | |  | **Model 3** | |
|  | **HR** **(95% CI)** | ***p*-value** |  | | **HR** **(95% CI)** | ***p*-value** |  | **HR** **(95% CI)** | ***p*-value** |
| Admission SI <0.6 | — |  |  | | — |  |  | — |  |
| Admission SI ≥0.6 | 2.32 (1.35 - 3.98) | 0.002 |  | | 2.25 (1.31 - 3.87) | 0.003 |  | 1.91 (1.10 - 3.33) | 0.022 |
| Model 1: Unadjusted.  Model 2: Adjusted for age and sex.  Model 3: Fully adjusted for LASSO-selected covariates including age, sex, Stanford classification, hypertension, diabetes mellitus, renal insufficiency, and anemia, and stratified by surgical intervention.  ***Abbreviations:*** *AD, aortic dissection; CI, confidence interval; HR, hazard ratio; SI, shock index.* | | | | | | | | | |
