# Supplementary figures and images for: Admission Shock Index Is an Independent Predictor of In‐Hospital All‐Cause Mortality in Patients With Acute Aortic Dissection and Intramural Hematoma
Source: Clin Cardiol. 2026 Apr 27;49(5):e70333. doi: 10.1002/clc.70333 (PMC13112594; doi:10.1002/clc.70333)

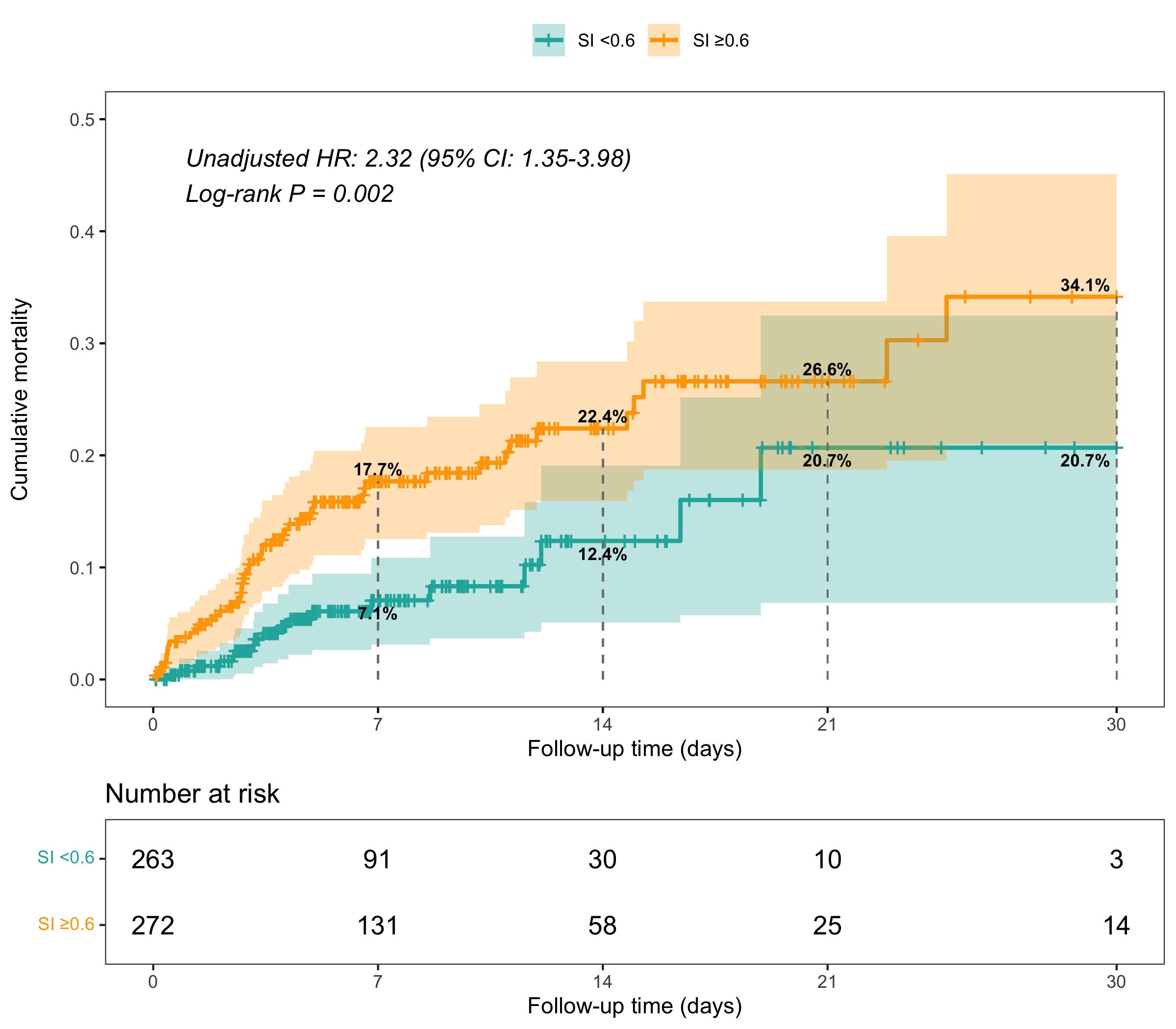

Supplement: Supplementary file 3 — Supporting File 3 [file CLC-49-e70333-s004.jpg]

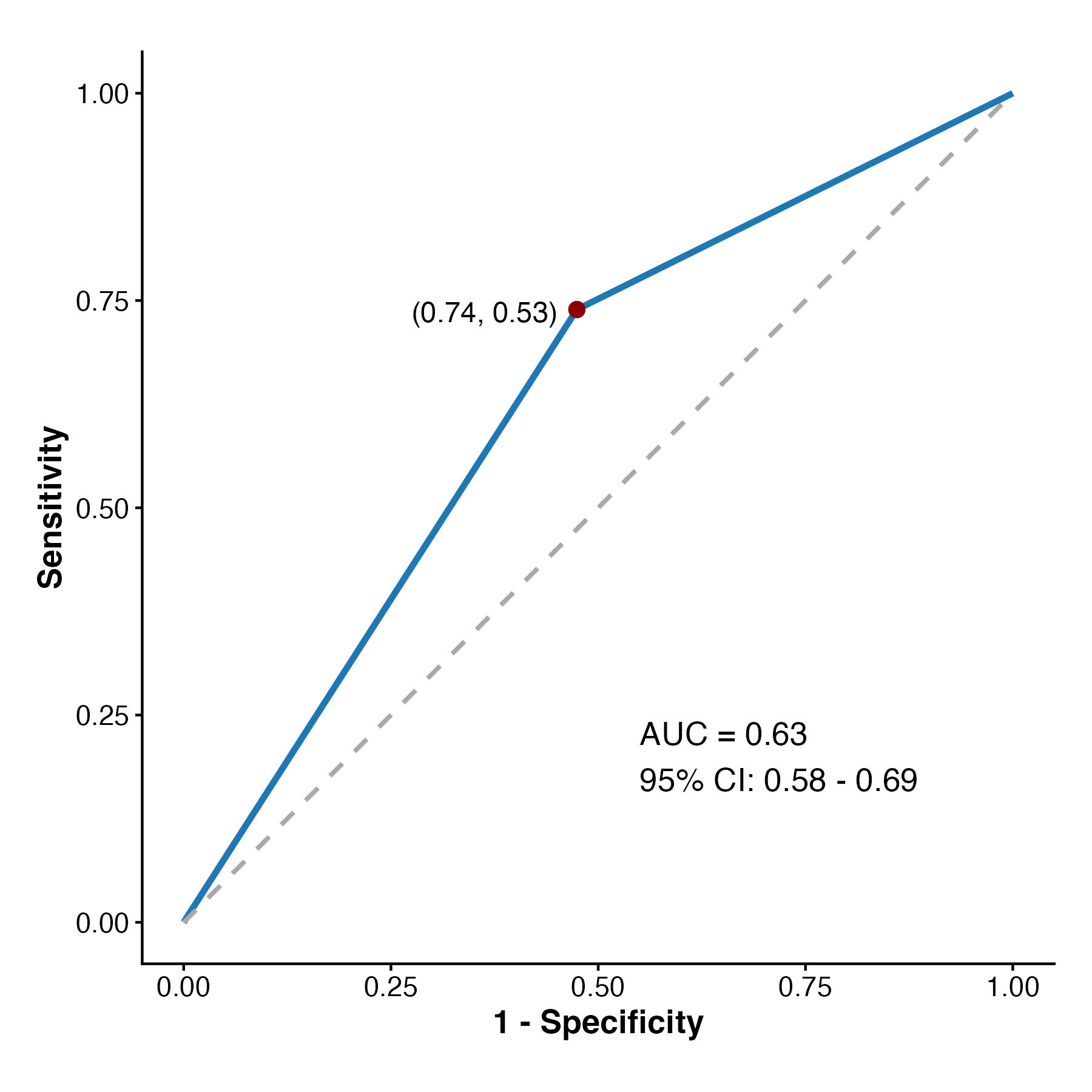

Supplement: Supplementary file 4 — Supporting File 4 [file CLC-49-e70333-s003.jpg]
